# Supplementary material for: The negligible role of carbon offsetting in corporate climate strategies
Source: Nat Commun. 2025 Sep 10;16:7963. doi: 10.1038/s41467-025-62970-w (PMC12423307; doi:10.1038/s41467-025-62970-w)
Supplement: Supplementary file 1 — Supplementary Information [file 41467_2025_62970_MOESM1_ESM.pdf]

The negligible role of carbon offsetting in corporate climate strategies

Niklas Stolz[1,*] and Benedict S. Probst[1,2,3]

[1]Group for Sustainability and Technology, ETH Zurich, 8092 Zurich, Switzerland
[2]Net Zero Lab, Max Planck Institute for Innovation and Competition, Germany
[3]Cambridge Centre for Environmental, Energy and Natural Resource Governance, University of Cambridge, UK
[*]nstolz@ethz.ch

Supplementary Information

Contents

S1. Influence of Inpex on Regression results **2**
Figure S1. Leave-on-out cross-validation 2
S2. Regression tables for scope 1 emission change and climate target ambition **4**
Table S1. 2023 and CDP 2018 survey waves as presented in Figure 1a. 4
Table S2: OLS Regression on corporate's climate targets ambition controlling for emission share covered by intermediate target as presented in Figure 1b. 5
S2. Sensitivity Analysis **5**
Table S3: OLS Regression on corporates' scope 1 emission ratio of CDP 2023 and CDP 2018 survey waves. 6
Table S4: OLS regression on corporate scope 1 and 2 emission performance 7
Table S5: OLS Regression on corporates' climate targets ambition 7
Table S6: OLS Regression on corporates' climate targets ambition controlling for estimated spending on carbon credits 9
Table S7: OLS Regression on corporates' climate targets ambition controlling for total number of carbon credits retired between CDP 2018 and CDP 2023 survey waves 9
Table S8: Logit Regression estimating the probability of corporates' intermediate climate targets to cover at least 50% of scope 1,2, and 3 emissions. 9
S3: Carbon credit costs in perspective **9**
Table S9: Estimated range of funds for European companies spent on retired carbon credits reported in the CDP 2023 survey compared to estimated spending on allowances on European Emission Trading Schemes (ETSs). Data presented in Figure 3. 9
References **10**

S1 Influence of Inpex on Regression results

We excluded Inpex Corporation from the regression evaluating the association between carbon credit retirements and scope 1 emissions since it is a strong outlier (Figure 1). While INPEX Corporation increased emissions by 1085% over the study time, the second largest emission increase was observed for Suncor Energy, with an increase of 81%. The increase in emissions is consistent with the CDP database and INPEX Corporation's sustainability reporting. However, due to the strong influence of the outlier on the statistical analysis, we exclude the company.

Figures S1a and b show that without INPEX Corporation, the only change in significance when leaving single observations out of the regression occurs for the categorical variable Oil and Gas sector, which is consistently close to the significance level. Both the continuous variable measuring the number of retired carbon credits during the CDP 2023 wave and the binary variable, which is 1 if companies retired more than 10,000 carbon credits and 0 otherwise, are robust to leaving single observations out.

When including INPEX Corporation, the estimates for both the continuous variable measuring retired carbon credits and the binary emission offsetting indicator are strongly changed. Figures S1b and c show the distribution of estimates when INPEX Corporation is included with the red marker indicating the single model run without INPEX Corporation. Including INPEX Corporation in the regression makes it seem like companies that offset more than 10,000 carbon credits (binary credit indicator) decarbonise significantly slower than companies that do not. However, this is not the correct conclusion due to the strong reliance on one data point and the robustness of results without the data point.

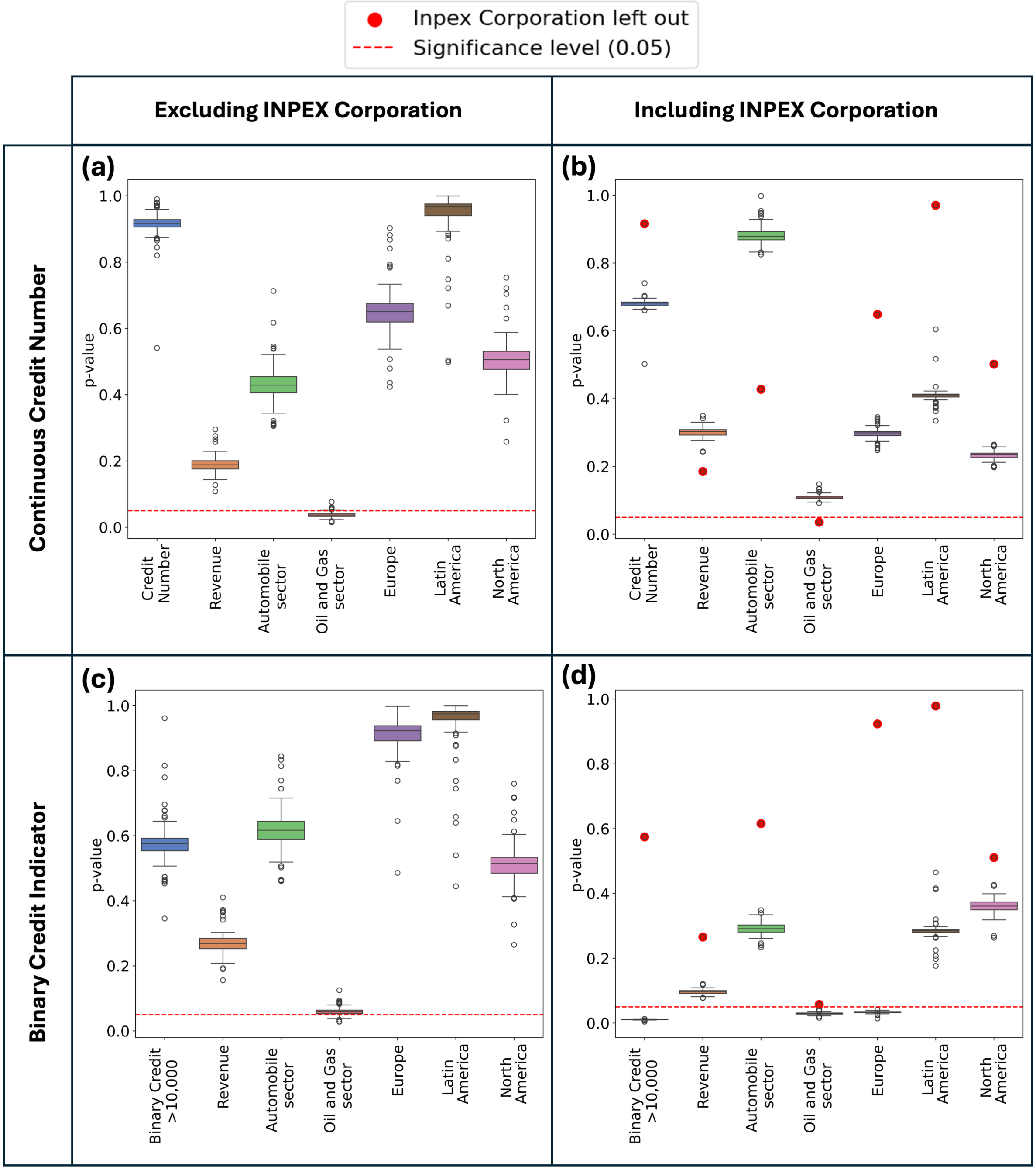

Figure S1. Leave-one-out cross-validation for the significance level of explanatory variables. The distribution represents the p-values of a two-sided test for each regression run ((a) and (c) n=77, (b) and (d) n=78 per regression run), with one company omitted in each iteration. The boxplots' center lines indicate the median p-value, the boxes span from the first quartile to the third, and the whiskers reach to the farthest point within 1.5x the inter-quartile range. In the left panels (a & c), the outlier INPEX Corporation is excluded from all regression runs. In the right panels (b & d), INPEX Corporation is included in all regression runs except for the points marked in red. In the top panels (a & b), the explanatory variable is the number of carbon credits a company retired during the CDP 2023 wave. In the bottom panels (c & d), the explanatory variable is a binary indicator set to 1 if a company retired more than 10,000 carbon credits and 0 otherwise.

S2 Regression tables for scope 1 emission change and climate target ambition

Table S1. Ordinary Least Squares regression on corporate's scope 1 emission ratio of CDP 2023 and CDP 2018 survey waves as presented in Figure 1a with standard errors in parentheses. Models 1-4 add control variables subsequently. Model 4 is presented in the main text. Asterisks indicate significance levels based on two-sided tests: * p<.1, ** p<.05, ***p<.01

	Model 1	Model 2	Model 3	Model 4
const	0.9412***	0.9412***	0.9412***	0.9412***
	(0.0313)	(0.0314)	(0.0304)	(0.0308)
Retired Carbon Credits	-0.0178	-0.0115	0.0055	0.0035
	(0.0313)	(0.0321)	(0.0325)	(0.0333)
Revenue		-0.0295	-0.0587*	-0.0495
		(0.0321)	(0.0350)	(0.0370)
Sector Automobile			0.0371	0.0343
			(0.0404)	(0.0430)
Sector Oil and Gas			0.0983**	0.0893**
			(0.0403)	(0.0416)
Europe				-0.0171
				(0.0374)
Latin America				-0.0013
				(0.0340)
North America				0.0251
				(0.0372)
R-squared	0.0042	0.0153	0.0969	0.1125
Adj. R-squared	-0.0089	-0.0110	0.0474	0.0238

Table S2. Ordinary Least Squares regression on corporate's climate targets ambition controlling for emission share covered by intermediate target as presented in Figure 1b with standard errors in parentheses. Models 1-5 add control variables subsequently. Model 5 is presented in the main text. Asterisks indicate significance levels based on two-sided tests: * p<.1, ** p<.05, ***p<.01

	Model 1	Model 2	Model 3	Model 4	Model 5
const	-10.0535***	-10.0535***	-10.0535***	-10.0535***	-10.0535***
	(0.6446)	(0.5790)	(0.5728)	(0.5179)	(0.4927)
Retried Carbon Credits	1.0834*	0.4375	0.2496	0.2208	0.1587
	(0.6446)	(0.5952)	(0.5993)	(0.5530)	(0.5303)
Intermediate coverage		2.7820***	2.6364***	1.6169**	0.8013
		(0.5952)	(0.5952)	(0.6172)	(0.6472)
Revenue			1.0038*	1.5586**	1.1999*
			(0.5936)	(0.6405)	(0.6211)
Sector Automobile				0.4076	0.3371
				(0.7057)	(0.6976)
Sector Oil and Gas				-2.3662***	-2.5644***
				(0.7693)	(0.7459)
Europe					1.7780***
					(0.6573)
Latin America					-0.4806
					(0.5550)
North America					-0.0214
					(0.5947)
R-squared	0.0314	0.2276	0.2528	0.4036	0.4798
R-squared Adj.	0.0203	0.2097	0.2264	0.3677	0.4277

S3 Sensitivity Analysis

We present sensitivity analyses for our findings around time range of carbon credit retirement, emission scope, presence of intensity target, funds spent on carbon credits, and probability that intermediate target exists. For each regression table we indicate in bold which variable is exchanged compared to the regressions presented in the main body.

Sensitivity analysis for carbon credit association with emission performance

Table S3. Ordinary Least Squares regression on corporates' scope 1 emission ratio of CDP 2023 and CDP 2018 survey waves. Compared to the main text, where only retired carbon credits in the CDP 2023 reporting period were used, here all credits retired between CDP 2018 and CDP 2023 survey waves enter an independent variable. Models 1-4 add control variables subsequently and standard errors are in parentheses. Asterisks indicate significance levels based on two-sided tests: * p<.1, ** p<.05, ***p<.01

	Model 1	Model 2	Model 3	Model 4
const	1.1050***	1.1050***	1.1050***	1.1050***
	(0.1568)	(0.1572)	(0.1563)	(0.1574)
**Retired Credits CDP 2018-2023**	-0.0343	-0.0165	0.0403	0.0534
	(0.1568)	(0.1585)	(0.1646)	(0.1676)
Revenue		-0.1363	-0.2183	-0.1637
		(0.1585)	(0.1736)	(0.1877)
Sector Automobile		-0.1363	-0.2183	-0.1637
		(0.1585)	(0.1736)	(0.1877)
Sector Oil and Gas			0.3231	0.2835
			(0.2144)	(0.2227)
Europe				-0.2306
				(0.2006)
Latin America				-0.1749
				(0.1755)
North America				-0.2401
				(0.1938)
R-squared	0.0008	0.0125	0.0552	0.0894
R-squared Adj.	-0.0151	-0.0193	-0.0078	-0.0224

Table S4. OLS Regression on corporates' scope 1 and 2 emission ratio of CDP 2023 and CDP 2018 survey waves. In the main text, only scope 1 emissions are used. Models 1-4 add control variables subsequently and standard errors are in parentheses. Asterisks indicate significance levels based on two-sided tests: * p<.1, ** p<.05, ***p<.01

	Model 1	Model 2	Model 3	Model 4
const	1.3024***	1.3024***	1.3024***	1.3024***
	(0.4055)	(0.4098)	(0.3979)	(0.4100)
Retired Carbon Credits	-0.1599	-0.0812	0.1452	0.2649
	(0.4055)	(0.4241)	(0.4300)	(0.5319)
Revenue		-0.3053	-0.9350	-0.8011
		(0.4241)	(0.5630)	(0.6917)
Sector Automobile			0.4970	0.5238
			(0.5104)	(0.5268)
Sector Oil and Gas			0.9626*	0.9611
			(0.5270)	(0.5538)
Europe				-0.6238
				(0.5574)
Latin America				-0.4056
				(0.4473)
North America				-0.4701
				(0.5331)
R-squared	0.0067	0.0296	0.1683	0.2492
R-squared Adj.	-0.0365	-0.0586	0.0020	-0.0599

Sensitivity analysis for carbon credit impact on climate target ambition

Table S5. OLS Regression on corporates' climate targets ambition controlling for emission share covered by intensity target. In the main text we controlled for the share of emissions covered by intermediate targets instead. Models 1-5 add control variables subsequently and standard errors are in parentheses. Asterisks indicate significance levels based on two-sided tests: * p<.1, ** p<.05, ***p<.01

	Model 1	Model 2	Model 3	Model 4	Model 5
const	-10.0535***	-10.0535***	-10.0535***	-10.0535***	-10.0535***
	(0.6446)	(0.6138)	(0.6030)	(0.5380)	(0.4970)
Retired Carbon Credits	1.0834*	0.4952	0.2431	0.3050	0.1671
	(0.6446)	(0.6414)	(0.6423)	(0.5840)	(0.5437)
**Share intensity target**		2.0247***	1.9380***	0.3340	0.2152
		(0.6414)	(0.6316)	(0.6628)	(0.6447)
Revenue			1.2555**	2.0524***	1.3419**
			(0.6197)	(0.6460)	(0.6242)
Sector Automobile				-0.0506	0.1734
				(0.7151)	(0.6903)
Sector Oil and Gas				-3.1449***	-2.8726***
				(0.8068)	(0.7610)
Europe					2.0699***
					(0.6184)
Latin America					-0.4887
					(0.5757)
North America					0.0544
					(0.6126)
R-squared	0.0314	0.1320	0.1720	0.3562	0.4705
R-squared Adj.	0.0203	0.1118	0.1428	0.3175	0.4176

Table S6. OLS Regression on corporates' climate targets ambition controlling for estimated spending on carbon credits. In the main text we controlled for the number of retired carbon credits. Models 1-5 add control variables subsequently and standard errors are in parentheses. Asterisks indicate significance levels based on two-sided tests: * p<.1, ** p<.05, ***p<.01

	Model 1	Model 2	Model 3	Model 4	Model 5
const	-10.0535***	-10.0535***	-10.0535***	-10.0535***	-10.0535***
	(0.6437)	(0.5790)	(0.5730)	(0.5179)	(0.4928)
**USD spent on credits**	1.1305*	0.4324	0.1997	0.2298	0.1322
	(0.6437)	(0.5983)	(0.6081)	(0.5626)	(0.5392)
Intermediate coverage		2.7748***	2.6439***	1.6120**	0.8020
		(0.5983)	(0.5972)	(0.6183)	(0.6478)
Revenue			1.0047*	1.5462**	1.2029*
			(0.5993)	(0.6478)	(0.6275)
Sector Automobile				0.4138	0.3328
				(0.7082)	(0.7002)
Sector Oil and Gas				-2.3668***	-2.5725***
				(0.7686)	(0.7453)
Europe					1.7780***
					(0.6578)
Latin America					-0.4811
					(0.5551)
North America					-0.0164
					(0.5941)
R-squared	0.0342	0.2275	0.2522	0.4036	0.4796
R-squared Adj.	0.0231	0.2095	0.2258	0.3677	0.4275

Table S7. OLS Regression on corporates' climate targets ambition controlling for total number of carbon credits retired between CDP 2018 and CDP 2023 survey waves. In contrast, in the main text only retired carbon credits in the CDP 2023 reporting period were used. Models 1-5 add control variables subsequently and standard errors are in parentheses. Asterisks indicate significance levels based on two-sided tests: * p<.1, ** p<.05, ***p<.01

	Model 1	Model 2	Model 3	Model 4	Model 5
const	-9.5690***	-9.5690***	-9.5690***	-9.5690***	-9.5690***
	(0.7162)	(0.6510)	(0.6490)	(0.5694)	(0.5416)
**Retired Credits CDP 2018-2023**	0.9531	0.4193	0.3364	0.2688	0.3782
	(0.7162)	(0.6647)	(0.6662)	(0.5976)	(0.5740)
Intermediate coverage		2.6513***	2.5323***	1.2826*	0.4321
		(0.6647)	(0.6700)	(0.6874)	(0.7213)
Revenue			0.7933	1.3636*	0.8591
			(0.6623)	(0.6898)	(0.6767)
Sector Automobile				0.5821	0.7590
				(0.7956)	(0.8009)
Sector Oil and Gas				-2.6403***	-2.6377***
				(0.8844)	(0.8443)
Europe					1.9124**
					(0.7589)
Latin America					-0.3590
					(0.6134)
North America					0.0291
					(0.6753)
R-squared	0.0243	0.2050	0.2212	0.4179	0.4971
R-squared Adj.	0.0106	0.1823	0.1874	0.3745	0.4342

Table S8. Logit Regression estimating the probability of corporates' intermediate climate targets to cover at least 50% of scope 1,2, and 3 emissions. In main text only Ordinary Least Squares regressions are presented. Models 1-4 add control variables subsequently and standard errors are in parentheses. Asterisks indicate significance levels based on two-sided tests: * p<.1, ** p<.05, ***p<.01

	Model 1	Model 2	Model 3	Model 4
const	1.6112	1.5685	1.7338	0.8513
	(1.0304)	(1.0556)	(1.4334)	(1.2600)
Retired Carbon Credits	7.6295	7.3327	7.6870	2.4448
	(4.7060)	(4.8493)	(6.5916)	(5.7514)
Revenue		0.1254	0.8539*	0.8499*
		(0.3092)	(0.4458)	(0.4670)
Sector Automobile			-0.6409*	-0.5604
			(0.3289)	(0.3817)
Sector Oil and Gas			-1.6668***	-1.8311***
			(0.3750)	(0.4344)
Europe				1.3774***
				(0.4660)
Latin America				0.1403
				(0.3037)
North America				0.4226
				(0.3460)
Pseudo R-squared	0.0948	0.0961	0.3233	0.4219

S4 Carbon credit costs in perspective

Copmany Name	Estimated emission offsetting costs (mln. USD)	Estimated ETS costs (mln. USD)	Ratio ETS costs / Upper Offsetting estimate	Scope 1 share covered by ETS (in %)
easyJet	17.3	172	9.9	80
Eni	11-31	1018	33.1	42.6
Shell	22-56	376	6.7	32.0
Volkswagen	17-27	231	8.6	65
Deutsche Lufthansa	1.8-3.6	206	56.7	31.8
Mercedes-Benz Group	3-5	19	4.1	95.7
Ryanair Holdings	1-2	602	352.2	83
Norwegian Air Shuttle ASA	0.075-0.08	41	496.4	89
BP	8-10	298	28.7	31
BMW Group	3-7	3	0.4	64

Table S9. Estimated range of funds for European companies spent on retired carbon credits reported in the CDP 2023 survey compared to estimated spending on allowances on European Emission Trading Schemes (ETSs). Data presented in Figure 3. Sources: Retired carbon credits and ETS allowances from the CDP database[1], average ETS prices from World Bank[2], Carbon credit price estimates - lower range from easyJet's annual report 2022, upper range from Ecosystem Marketplace (2023)[3]. Discrepancies between the reported ratio of ETS costs and upper offsetting estimate and reported absolute costs may arise since ratios are calculated with unrounded numbers.

References

1. CDP. CDP: Public Climate Change Dataset 2023 (2023).

2. World Bank Group: State and Trends of Carbon Pricing Dashboard (2023).

3. Ecosystem-Marketplace. 2023 State of the Voluntary Carbon Markets Report: Paying for Quality. Tech. Rep. (2023).
