# Supplementary material for: The negligible role of carbon offsetting in corporate climate strategies
Source: Nat Commun. 2025 Sep 10;16:7963. doi: 10.1038/s41467-025-62970-w (PMC12423307; doi:10.1038/s41467-025-62970-w)
Supplement: Supplementary file 2 — Description of Additional Supplementary Files [file 41467_2025_62970_MOESM2_ESM.pdf]

Description of Additional Supplementary Files

File Name: Supplementary Data 1
Description: The dataset contains all datapoints to re-create the figures in the manuscript. The sheet name indicates the figure number. To recreate the datapoints from raw data, please refer to the provided Github repository (see Code Availability).
