# Supplementary material for: The negligible role of carbon offsetting in corporate climate strategies
Source: Nat Commun. 2025 Sep 10;16:7963. doi: 10.1038/s41467-025-62970-w (PMC12423307; doi:10.1038/s41467-025-62970-w)
Supplement: Supplementary file 4 — Reporting Summary [file 41467_2025_62970_MOESM4_ESM.pdf]

nature portfolio

Corresponding author(s): Niklas Stolz

Last updated by author(s): 22.06.2025

Reporting Summary

Nature Portfolio wishes to improve the reproducibility of the work that we publish. This form provides structure for consistency and transparency in reporting. For further information on Nature Portfolio policies, see our Editorial Policies and the Editorial Policy Checklist.

Statistics

For all statistical analyses, confirm that the following items are present in the figure legend, table legend, main text, or Methods section.

n/a	Confirmed	
☐	☒	The exact sample size (n) for each experimental group/condition, given as a discrete number and unit of measurement
☐	☒	A statement on whether measurements were taken from distinct samples or whether the same sample was measured repeatedly
☐	☒	The statistical test(s) used AND whether they are one- or two-sided<br>*Only common tests should be described solely by name; describe more complex techniques in the Methods section.*
☐	☒	A description of all covariates tested
☐	☒	A description of any assumptions or corrections, such as tests of normality and adjustment for multiple comparisons
☐	☒	A full description of the statistical parameters including central tendency (e.g. means) or other basic estimates (e.g. regression coefficient) AND variation (e.g. standard deviation) or associated estimates of uncertainty (e.g. confidence intervals)
☐	☒	For null hypothesis testing, the test statistic (e.g. F, t, r) with confidence intervals, effect sizes, degrees of freedom and P value noted<br>*Give P values as exact values whenever suitable.*
☒	☐	For Bayesian analysis, information on the choice of priors and Markov chain Monte Carlo settings
☒	☐	For hierarchical and complex designs, identification of the appropriate level for tests and full reporting of outcomes
☒	☐	Estimates of effect sizes (e.g. Cohen's d, Pearson's r), indicating how they were calculated

Our web collection on statistics for biologists contains articles on many of the points above.

Software and code

Policy information about availability of computer code

Data collection	We used datasets provided by CDP who collect environmental data from companies annually.
Data analysis	We analysed the code using self written python code. For the statistical analysis we utilised the python package statsmodel (version 0.14.1). The requirements file in available code shows all used packages in code.

For manuscripts utilizing custom algorithms or software that are central to the research but not yet described in published literature, software must be made available to editors and reviewers. We strongly encourage code deposition in a community repository (e.g. GitHub). See the Nature Portfolio guidelines for submitting code & software for further information.

Data

Policy information about availability of data

All manuscripts must include a data availability statement. This statement should provide the following information, where applicable:

- Accession codes, unique identifiers, or web links for publicly available datasets
- A description of any restrictions on data availability
- For clinical datasets or third party data, please ensure that the statement adheres to our policy

The dataset used for the study are the CDP survey 2018 and 2019 (https://www.cdp.net/en)

All relevant data to reproduce plots can be found in our supplementary data and under https://github.com/n-stolnature_comms_negligible_role_carbon_offsetting.git. The data used in this article includes data points from CDP. The reproduction of any part of the CDP data by any third party is prohibited.

The data can be cited as: Niklas Stolz and Benedict Probst, The negligible role of carbon offsetting in corporate climate strategies, https://github.com/n-stolz/nature_comms_negligible_role_carbon_offsetting.git, DOI: 10.5281/zenodo.15634074, 2025.

Research involving human participants, their data, or biological material

Policy information about studies with human participants or human data. See also policy information about sex, gender (identity/presentation), and sexual orientation and race, ethnicity and racism.

Reporting on sex and gender	n/a
Reporting on race, ethnicity, or other socially relevant groupings	n/a
Population characteristics	n/a
Recruitment	n/a
Ethics oversight	n/a

Note that full information on the approval of the study protocol must also be provided in the manuscript.

Field-specific reporting

Please select the one below that is the best fit for your research. If you are not sure, read the appropriate sections before making your selection.

☐ Life sciences ☒ Behavioural & social sciences ☐ Ecological, evolutionary & environmental sciences

For a reference copy of the document with all sections, see nature.com/documents/nr-reporting-summary-flat.pdf

Behavioural & social sciences study design

All studies must disclose on these points even when the disclosure is negative.

Study description	We use a mixed-methods approach to evaluate the role of emission offsetting in corporate climate targets. First, we perform a ordinary least squares regression to evaluate whether there is a significant association between emission offsetting and emission reduction or climate target ambition; then, we explore quantitatively and qualitatively the reasons for the regression results.
Research sample	We use data on self-reported environmental data provided by CDP. This dataset is used by many studies evaluating corporate climate performance. The sample contains 25% of all carbon credits retired in 2022 and, therefore, a representative sample of emission offsetting companies in hard-to-decarbonise sectors.
Sampling strategy	Data for this study was obtained from the CDP (Carbon Disclosure Project) database, which collects environmental disclosure data from companies worldwide. Our focus was on large multinational companies within three specific sectors: aviation, automobile manufacturing, and oil and gas. These sectors were selected due to the prevalence of large-scale emission offset initiatives, which are central to the study's objectives.<br><br>We included all automobile manufacturers and airlines available in the CDP database to ensure comprehensive sector coverage. For the oil and gas sector, we sampled the top 40 companies by size and market influence. This cutoff was implemented to maintain comparability between oil and gas companies and those in the other sectors, thereby excluding smaller firms whose emission offset activities might differ substantially.
Data collection	Sustainability data was collected by CDP, a large data provider on self-reported sustainability data. Companies can choose to submit their data to CDP through CDPs online portal. Additionally we collected data on carbon credit usage from companies annual and sustainability reports. When companies retired >0 carbon credits in the 2023 CDP reporting wave, we downloaded sustainability and annual reports from their websites and searched the emission source they are offsetting. To find sections that talk about emission offsetting the researchers scanned the document for the words "offset*", "compensat*", "credit", "nature(-)based", "*forest*". Sections that discussed emission offsetting where coded using atlas.ti.
Timing	The sample includes companies' financial years ending between March 2022 and March 2023 for the CDP 2023 reporting wave and data that ended between March 2017 and March 2018 for the CDP 2018 reporting wave.
Data exclusions	We excluded PJSC Lukoil due to the heavy disruptions in the Russian oil and gas sector during the study. Further, we excluded Mercedes-Benz Group and INPEX Corporation for some statistical tests. the reasons are discussed in detail in the manuscript.
Non-participation	Companies can decide to report to CDP. However, since we did not conduct the sampling, we cannot definitively answer the

Non-participation	question. According to CDP, companies that represent two-thirds of the global market capitalisation disclose their data to them (https://www.cdp.net/en/press-releases/record-23-000-companies-disclose-environmental-impact-through-cdp-with-urgency-for-action-clear-in-wake-of-unprecedented-global-temperatures). Therefore, the coverage is very broad.
Randomization	We do not randomize in control group and control group since we want to test if there is a statistically significant difference between companies that decide to offset their emissions voluntarily and those that do not. Since the offsetting decision must be taken be the companies to be "voluntary", assignment to treatments by prescribing emission offsetting is not possible. Since we do not randomize into treatment and control group, we do not claim causal effects in the study.

Reporting for specific materials, systems and methods

We require information from authors about some types of materials, experimental systems and methods used in many studies. Here, indicate whether each material, system or method listed is relevant to your study. If you are not sure if a list item applies to your research, read the appropriate section before selecting a response.

Materials & experimental systems

n/a	Involved in the study
☒	☐ Antibodies
☒	☐ Eukaryotic cell lines
☒	☐ Palaeontology and archaeology
☒	☐ Animals and other organisms
☒	☐ Clinical data
☒	☐ Dual use research of concern
☒	☐ Plants

Methods

n/a	Involved in the study
☒	☐ ChIP-seq
☒	☐ Flow cytometry
☒	☐ MRI-based neuroimaging

Plants

Seed stocks	n/a
Novel plant genotypes	n/a
Authentication	n/a
